# Clinicopathological and Genomic Profiles of Atypical Fibroxanthoma and Pleomorphic Dermal Sarcoma Identify Overlapping Signatures with a High Mutational Burden

**DOI:** 10.3390/genes12070974

**Published:** 2021-06-25

**Authors:** Melike Ak, Abdullah Kahraman, Fabian M. Arnold, Patrick Turko, Mitchell P. Levesque, Martin Zoche, Egle Ramelyte, Reinhard Dummer

**Affiliations:** 1 Dermatology Department, University Hospital Zurich, 8091 Zurich, Switzerland; melike.ak@usz.ch (M.A.); patrick.turko@usz.ch (P.T.); mitchell.levesque@usz.ch (M.P.L.); egle.ramelyte@usz.ch (E.R.); 2 Faculty of Medicine, University of Zurich, 8006 Zurich, Switzerland; abdullah.kahraman@usz.ch (A.K.); fabian.arnold@usz.ch (F.M.A.); martin.zoche@usz.ch (M.Z.); 3 Pathology Department, University Hospital Zurich, 8091 Zurich, Switzerland

**Keywords:** atypical fibroxanthoma, pleomorphic dermal sarcoma, tumor genomic profiling, next-generation sequencing

## Abstract

Atypical fibroxanthoma (AFX) and pleomorphic dermal sarcoma (PDS) are rare tumors developing in chronically sun-exposed skin. Clinicopathological features are similar, but they differ in prognosis, while PDS has a more aggressive course with a higher risk for local recurrence and metastases. In current clinical practice, they are diagnosed by exclusion using immunohistochemistry. Thus, stringent diagnostic criteria and correct differentiation are critical in management and treatment for optimal outcomes. This retrospective single-center study collected clinicopathological data and tumor samples of 10 AFX and 18 PDS. Extracted genomic DNA from tumor specimens was analyzed by a next-generation sequencing (NGS) platform (FoundationOne-CDx™). Among 65 identified mutations, *TP53* inactivating mutations were observed in all tumor specimens. In both AFX and PDS, the known pathogenic gene alterations in *CDKN2A*, *TERT* promoter, and *NOTCH1* were frequently present, along with high mutational burden and stable Micro-Satellite Instability status. The mutational profiles differed only in *ASXL1*, which was only present in AFX. Further differences were identified in likely pathogenic and unknown gene alterations. Similarities in their genomic signatures could help to distinguish them from other malignancies, but they are not distinguishable between each other using the FoundationOne-CDx™ NGS panel. Therefore, histological criteria to determine diagnosis remain valid. For further insight, performing deep tumor profiling may be necessary.

## 1. Introduction

Atypical fibroxanthoma (AFX) and pleomorphic dermal sarcoma (PDS) are rare malignant cutaneous neoplasms of fibrohistiocytic mesenchymal origin. They both typically arise in chronically sun-exposed skin of elderly patients and show male predominance. AFX and PDS share similarities in clinicopathological features and their treatment; however, they differ in the disease course [1,2]. Thus, rigid diagnostic criteria are essential to ensure appropriate disease management and follow-up.

Clinically, AFX and PDS are both characterized as rapidly growing nodules. Both tumors can demonstrate ulceration and bleeding in the disease course. Histologically, both malignancies present proliferation of pleomorphic spindle and epithelioid cells or multinucleated giant cells, which often display atypical mitotic figures and hyperchromatism. Neoplastic cells are located under an occasionally thinned or ulcerated epidermis and extend into the reticular dermis. In contrast to AFX, PDS can display perineural and lymphovascular invasion, tumor necrosis, and a deeper infiltration into the subcutaneous tissue, muscle, or bone; hence, AFX is described as a superficial variant of PDS [1,3].

Both neoplasms are diagnosed by exclusion. Using immunohistochemistry (IHC), they can be differentiated from clinically and histologically similar tumors, such as spindle-cell squamous cell carcinomas (SCCs), melanoma, especially desmoplastic, and leiomyosarcoma. In contrast to the mentioned malignancies, neoplastic AFX and PDS cells stain positively for CD10, vimentin, α 1-antitrypsin and negative for Melan-A, S-100, SOX-10, cytokeratins, and desmin [1,3,4].

Consistent with clinical presentation in sun-exposed skin, previous studies revealed that most AFX and PDS harbor ultraviolet (UV)-induced (C > T or CC > TT) mutations in *TP53* and *TERT* promoter [5,6,7,8]. Moreover, frequent gene alterations in *CDKN2A*, *NOTCH1/2*, *COL11A1*, *FAT1* were reported in AFX and PDS, acting as possible drivers of proliferation [5,6,9,10]. Nonetheless, activating *RAS* mutation was observed in PDS but not in AFX, suggesting an extraordinary impact on the development of the more aggressive PDS [6,9].

Furthermore, a study evidenced *DNHD1*, *RTN1*, *RTL1*, *ZBTB7A*, *NCKAP5L*, and *FAM200A* as significantly mutated genes in PDS and demonstrated recurrent point mutation in *DDX31* (R72K) in five out of 28 PDS by performing whole-exome sequencing. Additionally, a high tumor mutational burden (TMB) with an average of 42.7 nonsynonymous variants per mega base was reported in the analyzed PDS cohort [11].

Treatment options for both malignancies are limited to surgery with wide local excision (WLE) or Mohs micrographic surgery (MMS). In AFX, MMS is considered as first-line treatment, as MMS results in a lower local recurrence rate of AFX in comparison to WLE [1,12]. Furthermore, treatment with WLE requires a surgical safety margin of two centimeters to reach a clearance of 96.6% AFX tumors, which is suboptimal for tissue conservation [13]. In PDS, the first-line treatment for optimal management is less defined. WLE with an excision margin less than two centimeters was reported to be a risk factor for tumor relapse in PDS [14]. While WLE with a minimum margin of one centimeter was described to have a recurrence rate of 3.7% in PDS [15]. No standard of care is established for AFX and PDS with unresectable, recurrent, or metastatic disease [3,16].

Even though AFX and PDS generally have a good prognosis, PDS is associated with a higher risk for local recurrence, resulting in up to 28% of patients [17] and distant metastases in up to 20% of the patients [18].

In this study, we aim to analyze the clinicopathological and genetic characteristics of AFX and PDS. Furthermore, we intend to improve the understanding of pathogenesis and seek to identify the molecular profile of AFX and PDS through next-generation sequencing (NGS) with the FoundationOne-CDx™ (F1CDx) platform [19]. Molecular profiles could improve the diagnostics and could help to distinguish AFX from PDS. Moreover, detected molecular alterations might lead to new treatment options such as targeted therapies.

## 2. Materials and Methods

In this retrospective study, we analyzed patients who fulfilled the following criteria: adult patients (≥18 years), diagnosed and/or treated for AFX and/or PDS at the Department of Dermatology, University Hospital Zurich, Switzerland, from January 2000 to December 2010 with a follow-up period of at least six months. Patients were excluded from the study if insufficient tumor material was available, or if the available samples had tumor content below 20%.

We identified the patients in the “DermaPro” (ifms GmbH, Saarbrücken, Germany) database by using the keywords “atypical fibroxanthoma, AFX, pleomorphic dermal sarcoma, dermal sarcoma NOS (not otherwise specified)”.

Tumor samples were histologically re-evaluated for diagnosis of AFX and PDS, tumor infiltration depth, and ulceration status. Tumor infiltration depth was measured in millimeters from the granular layer of the epidermis to the deepest point of invasion. In cases where tumor thickness was ≥1 mm and extended beyond the basal excision margin, tumor thickness was designated as ≥1 mm. Demographic patient data, as well as clinicopathological and histological features of the tumors, were obtained from electronic medical records.

**Figure 1:**
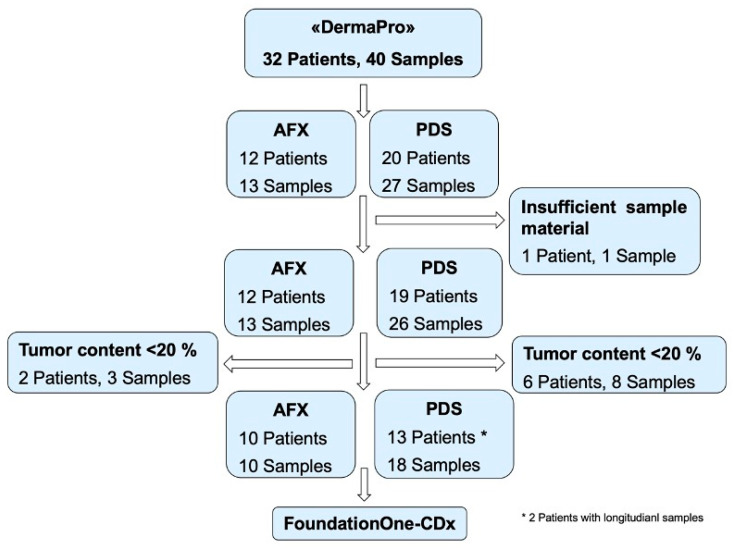
Representative flowchart of inclusion and exclusion of patients and samples. * 2 Patients with longitudinal samples.

Out of 32 identified patients, 23 were included in the final NGS analysis through F1CDx. Of 32 enrolled patients, we collected 40 Formalin-fixed paraffin-embedded (FFPE) tissue samples. More than one sample was available from five patients. One patient diagnosed with PDS was excluded from the study due to insufficient tumor material, and samples from eight patients (total 11 biopsies) with tumor content below 20% were also excluded from the study. In total, 10 samples from 10 patients diagnosed with AFX and 18 samples from 13 patients with PDS (one patient with four longitudinal samples within the timeframe of eight months, one patient with three longitudinal samples collected within nine months) underwent further analysis with NGS through F1CDx (Figure 1).

Total genomic DNA was extracted from FFPE tumor tissue samples with DNA isolation kits on a Promega Maxwell RSC. Isolated DNA and gene alterations were analyzed by a validated hybrid capture-based next-generation sequencing (NGS) platform (FoundationOne-CDx™) at the Department of Pathology and Molecular Pathology of the University Hospital Zurich, Switzerland. The methods of F1CDx have been previously described [19]. The current F1CDx gene panel (https://www.foundationmedicine.com (accessed on 29 November 2020)) includes cancer-related genes, 309 genes with full coding exons, 21 genes with selected intronic regions, one gene with a promotor region (*TERT*, telomerase reverse transcriptase), and one non-coding RNA gene (*TERC*, telomerase RNA component). Alterations were classified into known, likely and unknown pathogenicity by the F1CDx assay pipeline.

Additionally, Micro-Satellite Instability (MSI) and Tumor Mutational Burden (TMB) were assayed by F1CDx. TMB was measured as mutations (mt) per megabase (mb). TMB levels were divided into two groups: low (1–9 mt/mb) and high (≥10 mt/mb).

Patient primary tumor samples were stratified according to the histological diagnosis of AFX and PDS. For each tumor, the number of pathogenic mutations, variants of unknown significance, or both were counted per gene. In the case of multiple simultaneous mutations in a gene of a patient, the mutation count of the gene was incremented only once. The difference between the number of mutations of a gene in AFX and PDS was assessed by Fisher’s exact test using R (version 4.0.4). Multiple testing adjustment was made using Benjamini–Hochberg FDR (false discovery rate) correction. Differences in the TMB in the patient subgroups were analyzed through Wilcox–Rank sum test. A FDR adjusted *p*-value of <0.05 was considered statistically significant.

## 3. Results

### 3.1. Study Cohort

The AFX cohort included 10 patients with 10 representative AFX tumors, while the PDS cohort included 13 patients with 18 tumors. Five PDS tumor specimens represented local relapses and were obtained from two different patients with PDS.

The majority of patients with AFX (9/10, 90%,) and PDS (9/13, 69%) were male. The median age at diagnosis was 80.5 years (range: 61–90 years) in patients with AFX and 83.5 years (range: 71–92 years) in patients diagnosed with PDS. Two patients with PDS (15%) developed metastatic disease, with one patient developing nodal and one patient in-transit metastasis. No metastasis was observed in the AFX group. Local tumor relapse was present in one (10%) patient with AFX and three (23%) patients with PDS. Local relapses occurred after complete surgical excision, except for one patient with PDS who underwent incomplete excision.

### 3.2. Clinicopathological Features

**Figure 2:**
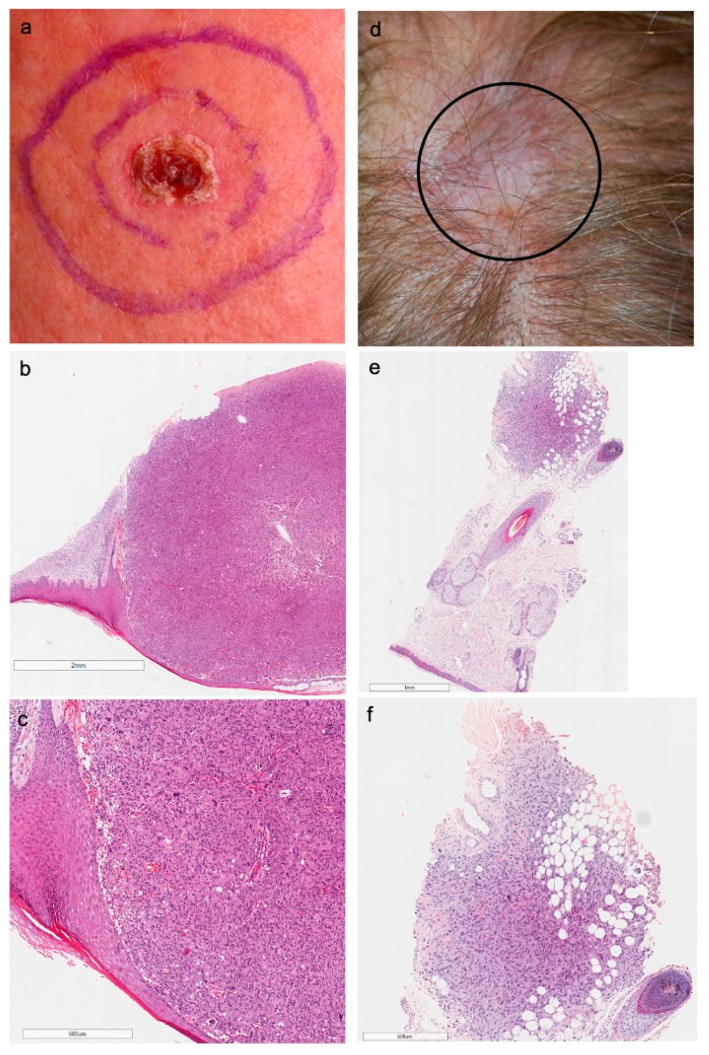
Clinical and histological picture of a male patient with AFX (**a**–**c**) and a female patient diagnosed with PDS (**d**–**f**). (**a**) AFX located at the vertex, (**b**) proliferation of pleomorphic spindle cells located under a thinned and ulcerated epidermis, (**c**) high power representation of atypical mitotic figures and multinucleated giant cells, (**d**) PDS located at the vertex, (**e**) neoplastic cells located at the deep dermis and infiltrating subcutaneous tissue, (**f**) pleomorphic, spindle-shaped neoplastic cells.

**Figure 3:**
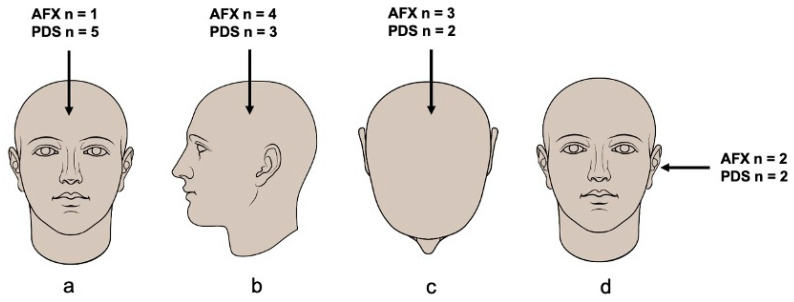
Primary tumor sites of AFX and PDS. (**a**) frontal area, (**b**) parietal area, (**c**) vertex, (**d**) auricular area. One primary PDS tumor was located at the lower leg is not represented in this figure.

Representative clinical and histological images of AFX and PDS from our cohort are shown in Figure 2. Most AFX and PDS primary tumors were located on the head (22/23, 96%), as represented in Figure 3. One (8%) PDS tumor was located in the lower leg. Baseline clinicopathological features of AFX and PDS of our study cohort are presented in Table 1. Upon IHC, neoplastic cells were generally negative for melanoma, cytokeratin, and muscle markers. In nine tumors, vimentin staining was used and showed positivity in 8 cases. CD10 staining was performed in two AFX tumors and was positive on neoplastic cells in both cases. (Supplemental Table S1).

### 3.3. NGS Analysis

To investigate the molecular landscape, we attempted to identify common and different driver events in primary tumors of the two patient subgroups by F1CDx analysis. Across 23 analyzed primary tumor samples a total of 65 mutations with known and likely somatic impact were identified. (Supplemental Table S2).

**Figure 4:**
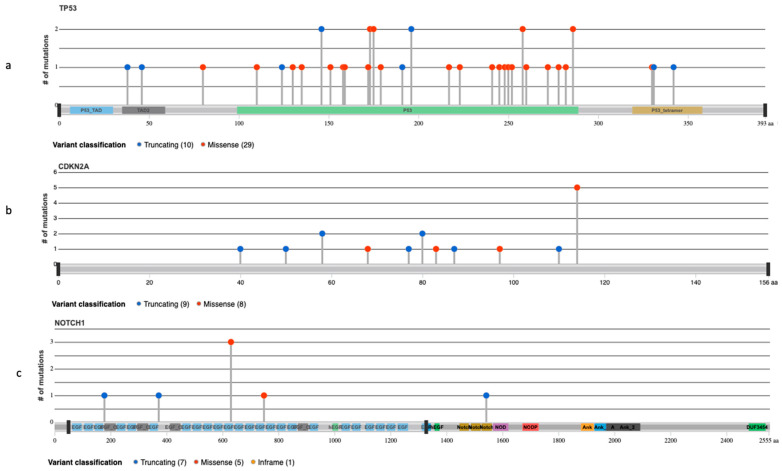
Lollipop plots highlighting genomic alterations of known and likely mutations in selected genes: (**a**) *TP53* (tumor protein 53), (**b**) *CDKN2A* (cyclin-dependent kinase inhibitor 2A), and (**c**) N*OTCH1* (NOTCH receptor 1) in AFX and PDS. Circles are colored with respect to the corresponding mutation types. Color codes are as follows: blue circles are indicating truncating mutations; red circles missense mutations and orange circles are indicating in-frame mutations. The height of the line represents the number of mutations at the specified position. The grey bar represents the entire protein with the different amino acid positions (aa). The coloured boxes are specific functional domains.

**Figure 5:**
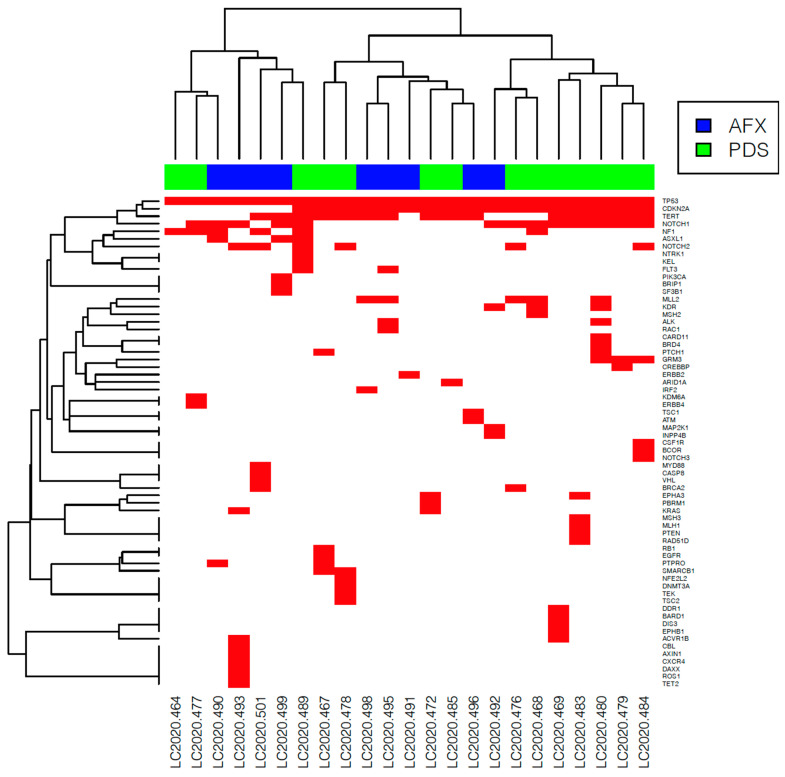
Heatmap of identified known and likely pathogenic gene alterations in atypical fibroxanthoma (AFX) and pleomorphic dermal sarcoma (PDS) tumor samples. The red box marks the presence of a gene alteration. The most frequent driver mutations are *TP53*, *CDKN2A*, *TERT* promoter, and *NOTCH1*.

All PDS and AFX tumors carried *TP53* (tumor protein 53) inactivating mutation (Figure 4a). A frequent driver gene evidenced in both subgroups was *CDKN2A* (cyclin-dependent kinase inhibitor 2A) gene loss or inactivating mutation, present in six (60%) AFX and 11 (85%) PDS samples (Figure 4b). Moreover, *NOTCH1* (NOTCH receptor 1) activating mutation was present in half (5/10) of the AFX and eight (62%) PDS tumors (Figure 4c). Further, *TERT* promoter activating mutation was frequently evidenced in six (60%) AFX and in nine (69%) PDS samples. However, no significant differences in the most frequent alterations were identified between the subgroups. (Figure 5 and Supplemental Table S3).

We have observed a non-statistically significant difference for *ASXL1* (additional sex combs-like 1) alteration between the subgroups. *ASXL1* mutation was detected in 3 AFX (30%) samples but in none of the PDS samples (*p* = 0.0593). (Supplemental Table S3).

Previously reported gene alterations in *FAT1* (FAT atypical cadherin 1) and *COL11A1* (collagen type XI α 1 chain)**,** *DNHD1* (Dynein Heavy Chain Domain 1), *RTN1* (Reticulon 1), *RTL1* (retrotransposon like 1), *ZBTB7A* (Zinc Finger And BTB Domain Containing 7A), *NCKAP5L* (NCK Associated Protein 5 Like), *FAM200A* (Family With Sequence Similarity 200 Member A) and *DDX31* (DEAD-Box Helicase 31) could not be assessed, as these genes were not part of the F1CDx panel. *KRAS* (Kirsten rat sarcoma) activating mutation was observed in one sample of each subgroup (AFX: 1/10, 10% and PDS: 1/13, 8%). Furthermore, we detected activating mutation in *NOTCH2* (NOTCH receptor 2) in a subset of our cohort (AFX: 3/10, 30% and PDS: 3/13, 23%).

Besides known and likely pathogenic mutations, we also analyzed mutations of unknown significance. A tendency for the difference in *GATA4* (GATA binding protein 4) alterations was observed among the subgroups, with half of the AFX (5/10) samples and one (8%) PDS sample demonstrated alteration in *GATA4* (*p* = 0.0501). Moreover, *MPL* (MPL proto-oncogene, thrombopoietin receptor), *PIK3CA* (phosphatidylinositol-4,5-bisphosphate 3-kinase catalytic subunit α), *CUL4A* (cullin 4A), and *DAXX* (death domain associated protein) were only present in the AFX subgroup and were observed in 3 (30%) tumors (*p* = 0.0593). One patient with AFX harbored all the above-mentioned alterations.

**Figure 6:**
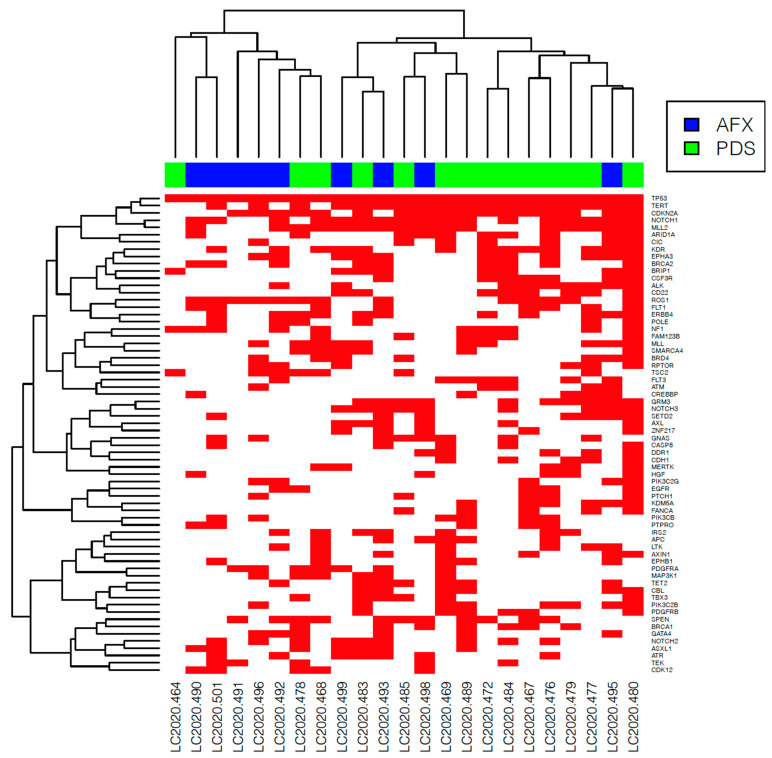
Heatmap of all identified gene alterations in the analyzed cohort. The red box marks the presence of a gene alteration.

In contrast, alterations in *CDH1* (cadherin 1) and in *CD22* (cluster of differentiation-22) were mostly observed in PDS samples. *CDH1* was evidenced in five (38%) PDS samples and was absent in the AFX subgroup (*p* = 0.053). Gene alteration in *CD22* were predominant in PDS (7/13, 54%) and present in one (10%) AFX sample (*p* = 0.0791). (Figure 6 and Supplemental Table S4).

**Figure 7:**
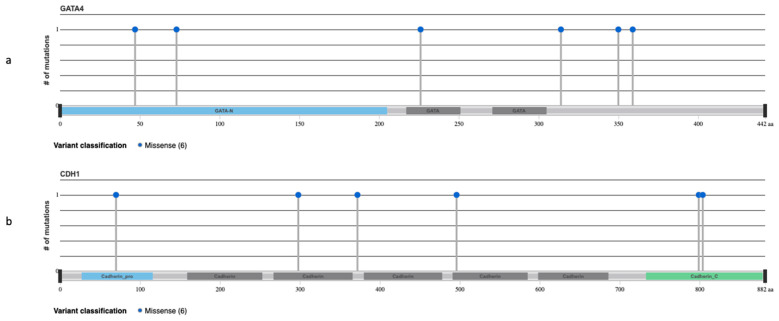
Lollipop plots highlighting genomic alterations of unknown mutations in selected genes: (**a**) *GATA4A* (GATA binding protein 4) and (**b**) *CDH1* (cadherin 1) in AFX and PDS. Blue circles are indicating truncating mutations. The height of the line represents the number of mutations at the specified position. The grey bar represents the entire protein with the different amino acid positions (aa). The coloured boxes are specific functional domains.

**Figure 8:**
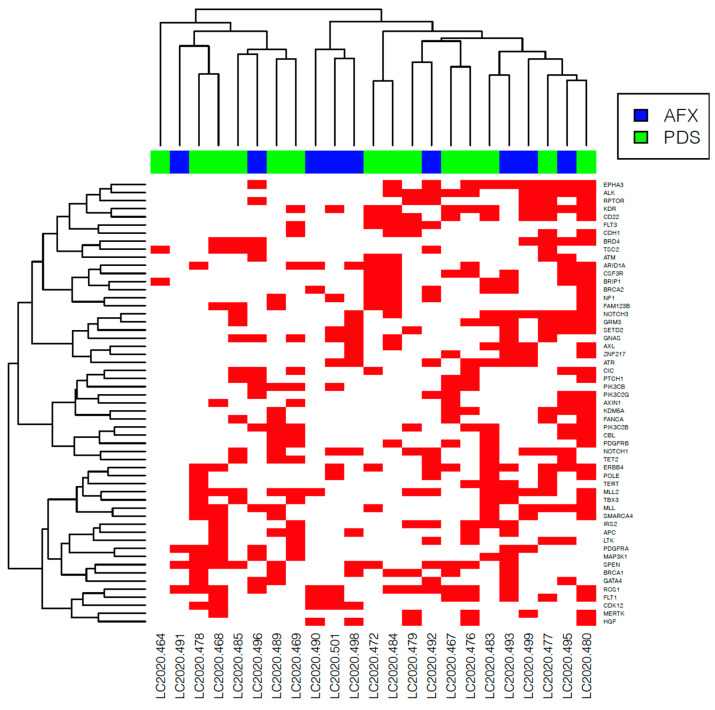
Heatmap of variants of unknown significance in atypical fibroxanthoma (AFX) and pleomorphic dermal sarcoma (PDS) tumor samples. The red box marks the presence of a gene alteration.

We further investigated the differences among subgroups based only on mutations of unknown significance. Matching tendencies in gene alteration were evidenced in *GATA4A* (AFX: 5/10, 50% and PDS: 1/13, 8%, *p* = 0.0501) (Figure 7a), *MPL* (AFX: 3/10, 30% and PDS: 0/13, *p* = 0.0593), *CUL4A* (AFX: 3/10, 30% and PDS: 0/13, *p* = 0.0593) alterations in AFX tumor samples and *CDH1* (AFX: 0/10, PDS: 5/13, 38% *p* = 0.053) (Figure 7b) alterations in PDS tumors. (Figure 8 and Supplemental Table S5).

*GNAS* (GNAS Complex Locus) was previously reported as a significantly mutated gene in PDS. In our analysis, alterations in *GNAS* were assigned in mutations of unknown significance and were evidenced in both subgroups (AFX: 4/10, 40% and PDS: 4/13, 30%, *p* = 0.6734).

Among all analyzed AFX and PDS tumors, including the longitudinal samples, most tumors (27/28, 96.4%) had high (≥10 mt/mb) TMB, while one PDS tumor had a low TMB (2.52 mt/mb). Mean measured TMB was 54.59 mt/mb in AFX and 69.49 mt/mb in PDS samples (*p* = 0.6484). MSI status was stable in all analyzed tumor samples. (Table 2 and Supplemental Table S6).

Among 18 PDS tumors, four longitudinal samples of one and three longitudinal samples of another patient with PDS underwent NGS analysis. Comparing recurrent specimens to primary PDS tumors among the same patient, we identified a consistency in the gene alterations. Most of the mutations showed a trend towards an increase in the variant allele frequencies. Additionally, a tendency to increase TMB was likewise observed.

## 4. Discussion

We have identified similarities in the demographic, clinicopathological, and genomic features of AFX and PDS. NGS analysis through F1CDx identified *TP53* inactivating mutation in all investigated primary AFX and PDS tumors. Known gene alterations in *CDKN2A*, *TERT*, and *NOTCH1* were frequently evidenced in AFX and PDS. Both subgroups harbored high TMB and had a stable MSI status. Differences between AFX and PDS were identified in likely and unknown gene alterations, although they were not statistically significant.

Our study included a cohort of 23 patients, highly representative for AFX and PDS cases considering their rarity among cutaneous malignant tumors. For AFX a prevalence rate of 0.24% was estimated in a retrospective study of over 42′000 skin tumors; however, the incidence of PDS and AFX is unknown [20]. The clinico-pathological characteristics of AFX and PDS in our cohort were as expected, showing consistency with previous reports [1,18].

Advances in NGS technology have encouraged clinicians to implement NGS analysis in guiding their daily clinical practice. This type of molecular profiling may provide insights into disease pathogenesis. Therefore, we screened for molecular signatures that could improve the distinction of AFX and PDS in addition to the current clinical practice.

NGS analysis of over 300 analyzed genes in the F1CDx panel, revealed high similarity in driver events (*TP53*, *TERT* promoter, *CDKN2A*, and *NOTCH1*), showing no significant difference between AFX and PDS. This is consistent with a previous report, where the authors pre-selected 11 genes for comparing the mutational profile of AFX and PDS [9].

Alterations in *TP53*, *TERT* promoter, *CDKN2A*, and *NOTCH1* have been previously reported in cSCC. cSCC, as well as AFX and PDS, are known to frequently develop in chronically sun-exposed skin areas. This could explain why they share similar UV- induced gene alterations in their molecular profile [21,22].

A difference in the subgroups among the known alterations was observed in *ASXL*, with a p-value above the significance level of 0.05 (*p* = 0.0593). Few AFX patients showed *ASXL1* alterations; whereas, these alterations have not been detected in patients with PDS. *ASXL1* is located on chromosome 20 and belongs to the group of ASX- like genes (ASXL1-3). It encodes a nucleoprotein which enhances regulatory proteins and plays a role in the stability of gene expression. *ASXL1* mutations have been previously observed in a variety of hematological malignancies in humans [23]. Even though *ASXL1* could help distinguish the respective subgroups, its occurrence is restricted to a subset of patients with AFX. Therefore, *ASXL1* cannot be used as an isolated distinct biomarker for AFX and explored in a larger cohort.

Including the unknown alterations as well as the known and likely mutations in our analysis, we noted potential differences in *GATA4*, *PIK3CA*, *CUL4A*, *DAXX*, *MPL*, *CDH1*, and *CD22* among AFX and PDS. Alterations of unknown significance have an unclear effect on function. However, these alterations have been previously reported in the context of various cancer entities, which may indicate biological relevance.

Half of the AFX cohort harbored a *GATA4* alteration of unknown significance. *GATA4* (chromosome 8) encodes for GATA-4, a member of the zinc finger transcription factors family (GATA-1-GATA-6). It is involved in the differentiation of endoderm- and mesoderm originated tissues and has been reported in various cancers such as gastrointestinal, ovarian, and lung cancer and glioblastoma multiforme [24,25]. Based on TCGA (The Cancer Genome Atlas) one unknown alteration evidenced in our AFX cohort (*GATA4 P359K*) has been previously reported in lung squamous cell carcinoma.

Alterations in *PIK3CA* were present in almost one-third of the AFX cohort (3/10, 30%). One mutation (E542K) has already been described in the literature [26]. On the contrary, *PIK3CA* mutations were absent in the PDS group. *PIK3CA* encodes p110-α, a subunit of phosphatidylinositol 3-kinase (PI3K). PI3K pathway is involved in cell signaling, which regulates cell functions such as cellular growth, proliferation, differentiation, and motility and was described as activating mutation in breast cancer [27,28]. In *PIK3CA*-mutated breast cancer targeted therapy with alpelisib, an α-selective phosphatidylinositol 3-kinase inhibitor is used for treatment [29]. Preclinical and clinical data in different tumor entities indicate that activating *PIK3CA* alterations may predict sensitivity to therapies targeting PI3K or AKT [30]. *PIK3CA*-mutated AFX tumors may therefore respond to mTOR inhibitors, including everolimus and temsirolimus, or an α-selective phosphatidylinositol 3-kinase inhibitor [31].

Comparable to *PIK3CA*, alterations in *CUL4A*, *DAXX*, and *MPL* were seen in 30% (3/10) of the AFX group and were not found in the PDS cohort. *CUL4A* (chromosome 13) is part of the cullin family. It acts as part of a ubiquitin-protein ligase complex and is associated with oncogenesis. Amplification and overexpression of *CUL4A* were reported in different tumor types (e.g., breast and prostate cancer, hepatocellular carcinoma) [32].

*DAXX* (chromosome 6) codes for death-associated protein 6. *DAXX* plays a role in tumorigenesis. Overexpression of *DAXX* has been reported to have an oncogenic function [33].

Myeloproliferative leukemia protein is encoded by *MPL* (chromosome 1). The role of activating *MPL* mutation has been reported in the context of myeloproliferative neoplasms [34].

On the contrary, our analysis revealed an opposite trend of alterations for *CDH1* and *CD22* within the explored neoplasms. *CDH1* was only present in PDS (5/13, 38%). Inactivating mutation of *CDH1* (chromosome 16), encoding for E-cadherin, are known to contribute to malignant cell detachment from the primary tumor [35,36].

Additionally, *CD22* (chromosome 19) was evidenced in more than half of the PDS cohort and one AFX tumor. *CD22* encodes for a surface molecule and is known to be expressed by most blasts of B-cell acute lymphoblastic leukemia [37].

Altogether, all listed alterations are known to play a role in cancer. However, alterations with unknown somatic impact are still poorly investigated in the context of AFX and PDS and require further in-depth studies. Moreover, the difference in the listed alterations among AFX and PDS was not significant. Thus, they are not suitable as potential biomarkers to discriminate the explored malignancies.

Our results showed that AFX exhibits high TMB, also known as a mutational load. The PDS subgroup also evidenced high TMB, which is in line with a previous report [11]. TMB is affected by different sources, including exposure to mutagenic agents such as ultraviolet light in melanoma and cigarette smoke in lung cancer [38,39]. Hence, we could hypothesize that the investigated neoplasms harbor high TMB due to chronic UV- exposure, given that they arise in high sun-exposed skin areas. A low TMB of 2.52 mt/mb was noted only in one biopsy taken from the lower leg, a site less chronically exposed to the sun.

In comparison to cSCC TMB levels were reported equally high (median TMB 45.2 mt/mb) [40]. In other cutaneous sarcoma subtypes, such as superficial angiosarcoma, high TMB (20.7 mt/mb) was lower than observed in our cohort [41].

High TMB is known to be associated with a better response to immunotherapy with checkpoint inhibitors (CPI) such as anti-PD-1 and anti-PD-L1 across diverse tumor entities [42]. Interestingly, a case study reported that two patients diagnosed with advanced PDS and high TMB have been successfully treated with anti-PD-1 (Pembrolizumab, (KEYTRUDA^®^ MSD Kenilworth, NJ, USA). One patient with a locally metastasized PDS (TMB 63.162 mt/mb) showed complete remission after 8 cycles of treatment with Pembrolizumab. Another patient with an inoperable relapsed PDS (TMB 77.997 mt/mb) treated with Pembrolizumab for four months, similarly showed a complete response. However, this patient received additional radiotherapy, introducing an additional factor potentially influencing treatment response [42]. Pembrolizumab is approved for other UV-induced skin tumors, such as malignant melanoma [43]. Therefore, we suggest that metastasized and/or unresectable AFX and PDS could be considered for immunotherapy with CPIs.

## 5. Conclusions

Our analysis highlights that mutations in *TP53*, *TERT* promotor, *CDKN2A*, and *NOTCH1* are frequent in AFX and PDS, along with a high TMB. This signature could help to distinguish AFX and PDS from other malignancies, although this molecular profile is not unique for AFX and PDS.

Our data demonstrate that AFX and PDS are not distinguishable from each other with the current F1CDx panel. Thus, our findings did not reveal new rigid diagnostic criteria to distinguish AFX from PDS. The current clinical practice using histological criteria to determine the diagnosis and the prognosis remains valid. Nevertheless, an interesting trend in *ASXL1* occurring more frequently in AFX has been noted. To further investigate the prevalence of *ASXL1* in AFX, NGS analysis of a larger cohort is necessary. The inclusion of somatic alterations with unknown impact into our analysis showed a potential difference in *GATA4*, *PIK3CA*, *CUL4A*, *DAXX*, *MPL*, *CDH1*, and *CD22* among the subgroups. Further studies with the inclusion of the unknown alterations may present significant differences between AFX and PDS. For further insight into the molecular pattern of the investigated malignancies, performing deep tumor profiling such as single-cell analysis may be required. This could identify new biomarkers unique for AFX or PDS. Furthermore, our data show that AFX and PDS harbor high TMB, which is expected from a UV-exposed anatomical site. Therefore, unresectable and/or metastasized AFX and PDS harboring high TMB could be considered for immunotherapy. Although immunotherapy could offer a new promising therapeutic approach, further investigations in randomized prospective trials of immunotherapy in AFX and PDS patients with high TMB are needed.

## Figures and Tables

**Table 1:** Clinicopathological features of atypical fibroxanthoma (AFX) and pleomorphic dermal sarcoma (PDS).

	AFX	PDS	Total
	(*n* = 10)	(*n* = 13)	(*n* = 23)
Diameter (cm)			
≤2	3	2	5
2.1–3.9	0	0	0
≥4.0	1	0	1
Unknown	6	11	17
Invasion depth (mm)			
≥1	3	5	8
≤2	3	0	3
2.1–3.9	1	5	6
≥4.0	3	3	6
Ulceration			
Present	5	7	12
Absent	5	6	11

**Table 2:** Tumor Mutational Burden (TMB) and Micro-Satellite Instability (MSI) status of all analyzed tumor samples.

	AFX	PDS	Total
	(*n* = 10)	(*n* = 18)	(*n* = 28)
TMB-Status			
High (≥10 mt/mb)	10	17	27
Low (1–9 mt/mb)	0	1	1
TMB (mt/mb)			
Mean	54.59 (20.2–90.8)	69.49 (2.52–157.2)	64.17 (2.52–157.2)
MSI-Status			
Stable	10	18	28

## Data Availability

The data presented in this study are available in Supplemental Tables S1–S6 files.

## Supplementary Materials

The following are available online at https://www.mdpi.com/article/10.3390/genes12070974/s1. Table S1: IHC features of AFX and PDS tumor samples, Table S2: Gene alterations identified by F1CDx, Table S3: Results of statistical analysis of known and likely gene alterations, Table S4: Results of statistical analysis of all identified gene alterations, Table S5: Results of statistical analysis of unknown gene alterations, Table S6: TMB and MSI-Status identified by F1CDx.
