# Supplementary material for: Clinicopathological and Genomic Profiles of Atypical Fibroxanthoma and Pleomorphic Dermal Sarcoma Identify Overlapping Signatures with a High Mutational Burden
Source: Genes (Basel). 2021 Jun 25;12(7):974. doi: 10.3390/genes12070974 (PMC8303615; doi:10.3390/genes12070974)
Supplement: Supplementary file 1 [file genes-12-00974-s001.zip › Supplemental_Table_S1_IHC.pdf]

	AFX (n= 10)	PDS (n= 13)	Total (n= 23)
**AE1**			
Positive	1	0	1
Negative	6	10	16
Unknown	3	3	6
**p63**			
Positive	0	0	0
Negative	5	3	8
Unknown	5	10	15
**Pan-Cytokeratin**			
Positive	0	0	0
Negative	1	1	2
Unknown	9	12	21
**CK7**			
Positive	0	0	0
Negative	0	1	1
Unknown	13	12	25
**HMB45**			
Positive	0	0	0
Negative	2	2	2
Unknown	8	11	21
**Melan-A**			
Positive	0	0	0
Negative	6	9	15
Unknown	4	4	8
**s-100**			
Positive	1	0	1
Negative	7	11	18
Unknown	2	2	4
**SOX-10**			
Positive	0	0	0
Negative	1	0	1
Unknown	9	13	22
**Tyrosinase**			
Positive	0	0	0
Negative	0	3	3
Unknown	10	10	20
**ki-67**			
Positive	5	4	9
Negative	0	0	0
Unknown	5	9	14
**CD31**			
Positive	1	1	2

Negative	2	2	4
Unknown	7	10	17
**FXIII-A**			
Positive	1	2	3
Negative	0	1	1
Unknown	9	10	19
**CD117**			
Positive	0	0	0
Negative	0	1	1
Unknown	10	12	22
**CD34**			
Positive	2	0	2
Negative	1	6	7
Unknown	7	7	14
**Actin**			
Positive	0	1	1
Negative	0	1	1
Unknown	10	11	21
**Desmin**			
Positive	0	0	0
Negative	3	3	6
Unknown	7	10	17
**alpha SMA**			
Positive	0	0	0
Negative	1	4	5
Unknown	9	9	18
**Podoplanin**			
Positive	0	0	0
Negative	0	2	2
Unknown	10	11	21
**Lysozme**			
Positive	0	0	0
Negative	0	1	1
Unknown	10	12	22
**CD30**			
Positive	0	0	0
Negative	0	1	1
Unknown	10	12	22
**CD68**			
Positive	1	7	8
Negative	2	1	3
Unknown	7	5	12
**CD20**			
Positive	0	0	0

Negative	0	1	1
Unknown	10	12	22
**CD10**			
Positive	2	0	2
Negative	0	0	0
Unknown	8	13	21
**Vimentin**			
Positive	4	4	8
Negative	1	0	1
Unknown	5	9	14
**MITF**			
Positive	0	0	0
Negative	1	1	2
Unknown	9	12	21
**CEA**			
Positive	0	0	0
Negative	0	1	1
Unknown	10	12	22
**EMA**			
Positive	0	0	0
Negative	0	3	3
Unknown	10	10	20
**GLUT-1**			
Positive	0	0	0
Negative	0	1	1
Unknown	10	12	22

Supplemental Table S1. Immunhistochemical features of AFX and PDS tumor samples
